# Validation of a New Test for Measuring the Contrast Sensitivity Function (Optopad-CSF) at Near Vision

**DOI:** 10.3390/diagnostics14131377

**Published:** 2024-06-28

**Authors:** Dolores de Fez, Celia García, Maria Josefa Luque-Cobija, Kevin J. Mena-Guevara, Paula Daudén, David P. Piñero

**Affiliations:** 1 Psychophysics and Visual Perception Group, Department of Optics, Pharmacology and Anatomy, University of Alicante, 03690 San Vicente del Raspeig, Spain; c.garcia@ua.es (C.G.); kjmg92@gmail.com (K.J.M.-G.); david.pinyero@ua.es (D.P.P.); 2 Department of Optics, Optometrý and Visual Sciences, University of Valencia, Burjassot, 46100 Valencia, Spain; maria.j.luque@uv.es (M.J.L.-C.); pauladaudenoliver@gmail.com (P.D.)

**Keywords:** achromatic contrast sensitivity function, Optopad Project, Optopad-CSF, CSV-1000E, cataracts

## Abstract

Our purpose is to develop and validate a new iPad-based contrast sensitivity (CS) test for measuring the contrast sensitivity function at near vision (Optopad-CSF). A total of 200 eyes of 100 healthy subjects (ages 17–63) were evaluated in a comparative study between the Optopad-CSF test (near vision) and the CSV-1000E test (distance vision). The agreement between tests was assessed with the index of contrast sensitivity (ICS) and the area under the curve (AUC). CS for all the spatial frequencies in both eyes showed a negative significant correlation with age, and corrected distance, and near visual acuities (r ≤ −0.512, *p* ≤ 0.013). A significantly lower CS was found with the Optopad-CSF test in the over-40-year-old subgroup for all the spatial frequencies evaluated compared to the below-40 subgroup (*p* ≤ 0.008). The mean AUC of the Optopad-CSF test (5.84) was twice that of the CSV-1000E test (2.76). The mean ICS of the Optopad-CSF (−0.019) and CSV-1000E (−0.075) tests showed similar values, both close to 0 (*p* = 0.3). There was a weak but significant correlation between the Optopad-CSF and CSV-1000E ICS tests (r = 0.246, *p* < 0.02). A range of normality for the values obtained with the Optopad-CSF test was calculated. The mean CS values in 16 bilateral cataract patients were out of the normal range for all the spatial frequencies evaluated (*p* < 0.001). Optopad-CSF is a valid portable system for measuring CS at near vision for five spatial frequencies, allowing the detection of age-related changes in CSF with age and CSF loss in cataracts, with no ceiling effect.

## 1. Introduction

According to the World Health Organization, approximately 90 million people suffer from vision impairment or blindness in the European region. The most common eye conditions include refractive errors, such as myopia, cataracts, age-related macular degeneration, diabetic retinopathy, and glaucoma. Uncorrected refractive errors and cataracts are the leading causes of vision impairment in the European region [1].

There is a wide range of symptomatology associated with vision impairment, including decreased ability to recognize faces and objects and loss of color vision. These symptoms interfere with performing daily activities such as driving, reading, or working with the computer. The detection and characterization of losses in contrast sensitivity, chromatic sensitivity, visual acuity, or other visual and oculomotor abilities allows the clinician to detect and prevent the progression of some anomalies as well as determine measures to be implemented for the improvement of the patients’ quality of life.

Contrast sensitivity represents the ability of the visual system to detect small differences in contrast. Its relevance in visual perception is related to the perception of shapes and spatial details. The measurement of the contrast sensitivity function (CSF) reveals the decrease that the visual system makes in the contrast of achromatic gratings, depending on their frequency, that is, for different sizes and details of objects. This decrease is like that carried out by any conventional optical system, except for the fall at low frequencies, which is due to the neural part of the visual system. Contrast sensitivity is one important aspect of visual function that can be characterized in clinical practice. Specifically, the clinical measurement of the CSF can currently be performed through printed tests (e.g., VCTS—Vistech Consultants, Inc., Dayton, OH, USA—FACT), retro-illuminated tests (e.g., CSV-1000E), or using a part of a vision tester not specifically oriented to CSF measurement (e.g., Metropsis, Functional Vision Analyzer, Metrovision, Topcon LED LCD chart system, etc.). However, oftentimes, researchers and clinicians cannot acquire a commercial version of these tools for CSF measurement because they are not currently manufactured or due to the high cost of some vision testers.

Digital developments in recent years have made possible the creation of computer- or tablet-based versions of classic printed visual tests, such as HACSS [2], OCST [3], or ClinicCSF [4]. This digital transformation of clinical contrast sensitivity tests may seem like a simple procedure, but the change from a printed to a screen-based version of a CSF test is not an easy and immediate task. There are some digital tests that use the physical characteristics of the stimuli that are based on the generic properties of a device type (e.g., resolution, gamma curve of luminance). However, our group has conducted several studies showing that this procedure can lead to diagnostic errors that are not clinically acceptable [5,6,7]. It is necessary to know the spatial resolution and color reproduction characteristics of each specific display; otherwise, the reliability of the test may be compromised, with displays that do not really show the specific stimuli characteristics (f.i., mean luminance, contrast, or spatial frequency) that have been intended [6,7,8].

Besides the potential problems of stimulus reproduction in contrast sensitivity tests, there is another critical aspect that must be considered. Generally, the measurement conditions are not strictly comparable, especially when comparing printed tests, autoluminescent devices, or displays. The main factors that can modify the values of contrast sensitivity measurements are viewing distance, light source, intensity (background and mean luminance of the stimulus), stimulus size, contrast values, spatial frequencies, and the psychophysical method. Experimental results from different studies have shown the difficulty of performing comparisons between tests as well as establishing a gold standard for the measurement of the CSF [9,10,11,12,13,14,15,16].

In the present study, our group has used a prototype of a novel digital platform for visual function assessment, the Optopad Project [17,18,19]. This platform groups a set of visual tests designed for low-cost portable visualization devices which evaluate the chromatic and achromatic visual mechanisms. The design of each test includes the particular colorimetric profile of the screen where the stimuli will be displayed. The colorimetric characterization process of the Optopad platform, as well as the validation and results of clinical studies of a test that evaluates color discrimination (Optopad-Color), have been described in detail in previous research studies [5,6,17,20]. In the current study, a specific test that only evaluates the achromatic CSF (Optopad-CSF) has been used.

The aim of this study was to validate the results of the Optopad-CSF test by studying its agreement with a widely used test (CSV-1000E [21]), by confirming that the relationship between contrast sensitivity and age in both tests is consistent with the literature, and by measuring a group of patients with significantly decreased contrast sensitivity in order to check if the Optopad-CSF test could detect their visual losses. In addition, the normal range for clinical use of this new test has been determined.

## 2. Materials and Methods

### 2.1. Patients

Healthy subjects were recruited consecutively from those attending the Optometric Clinic of the University of Alicante. Only subjects without any active systemic or ocular condition were included. Subjects were excluded in case of previous ocular surgery, neurologic diseases or previous eye diseases such as uveitis, cataract, retinal problems, or any pathology affecting corneal transparency. All patients were informed about the nature of the study before their inclusion and provided written informed consent according to the tenets of the Declaration of Helsinki. The study was approved by the ethics committee of the University of Alicante (UA-2021-10-15_2). Besides this sample of eyes, another sample of patients with bilateral cataracts was included. Inclusion criterion was presence of bilateral cataract, at least NO3 or C4, according to the LOCS III grading system. Exclusion criteria were previous ocular surgery and any other active ocular disease (including retinal diseases) that could affect contrast sensitivity measures.

### 2.2. Examination Protocol

All experimental measurements were performed by the same examiner in the normal group (P.D.) and in the cataractous group (K.J.M-G.) A complete eye exam was performed that included measurement of uncorrected (UDVA) and corrected distance visual acuity (CDVA), manifest refraction and near addition, measurement of uncorrected (UNVA) and corrected near visual acuity (CDVA), measurement of distance-corrected near visual acuity (DCNVA), analysis of ocular alignment with the cover test, evaluation of the integrity of the anterior and posterior ocular segment with the slit lamp biomicroscopy, characterization of ocular parameters including pupillometry, anterior chamber depth (ACD), corneal curvature and asphericity, and ocular aberrations with the VX120 multidiagnostic system (Visionix-Luneau Technologies, Chartres, France), and measurement of the axial length (AXL) with the IOL-Master system (Carl Zeiss Meditec, Jena, Germany).

Contrast sensitivity measurements were performed with two devices: the commercial test CSV-1000E (VectorVision, Greenville, OH, USA) and the prototype of the Optopad-CSF test (1.0 version), not yet commercially available. This device is part of the results obtained in the Optopad Project (University of Alicante-University of Valencia [19]).

### 2.3. Contrast Sensitivity Measurements

**Figure 1:**
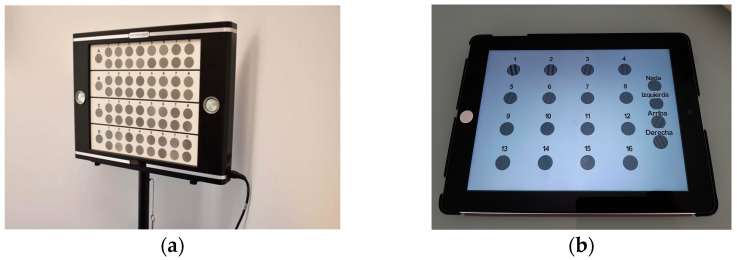
(**a**) The CSV-1000E test, placed at 2.5 m from the patient, on a stand or directly on a wall. (**b**) iPad with Optopad-CSF test, placed 40 cm from the patient. Both tests are placed perpendicular to the observer.

CSV-1000E measures the contrast sensitivity function by a forced-choice, descending limits method, using sinusoidal gratings of 3, 6, 12, and 18 cycles per degree (cpd), and 8 levels of decreasing contrast (see Figure 1a). The test is divided into four translucent charts, one for each frequency, with a background illumination (85 cd/m^2^) provided by a non-polarized fluorescent luminance source.

On each chart, the stimuli are displayed in a double row of circles, so that for each contrast, the stimulus only appears in one of the two possible positions (top or bottom, randomly) for the patient to select which one contains the stimulus (two-alternative forced choice paradigm). The sensitivity values for each stimulus are frequency-dependent. The normality values were taken from the literature [22], selecting the range of 22 to 55 years, which most closely matches our population.

Optopad-CSF 1.0 is a fast and non-invasive method to characterize the CSF at near distance on a portable electronic display device emitting polarized light (Apple iPad 6th Gen A1893, see Figure 1b). The iPad retina screen has a display size of 2048 × 1536 pixels at 267 pixels per inch, with a screen size of 9.7 in and 8-bit per channel color resolution. To correctly reproduce the spatial and colorimetric characteristics of the designed stimuli, the device was previously colorimetrically characterized using the 3DLUT method [5]. See a more extensive description in the following section.

Measurements were performed in a dark room, monocularly, after 3 min of adaptation to the observation conditions and at a distance of 2.5 m for the CSV-1000E and 40 cm for the Optopad-CSF (with the best correction in each case). Both eyes of each patient were explored.

### 2.4. Optopad-CSF Test Design and Experimental Setup

The device is designed to measure spatial contrast sensitivity function with stimuli modulated along the cardinal directions of the DKL space [23] (A:Achromatic, T:Red-Green, D:Blue-Yellow) defined from the device’s achromatic stimulus with mean luminance of 60 cd/m^2^. In this study, only the plates corresponding to the achromatic were used in order to be able to compare them with the results obtained with the CSV-1000E test, and the Michelson contrast values were used in both tests.

**Figure 2:**
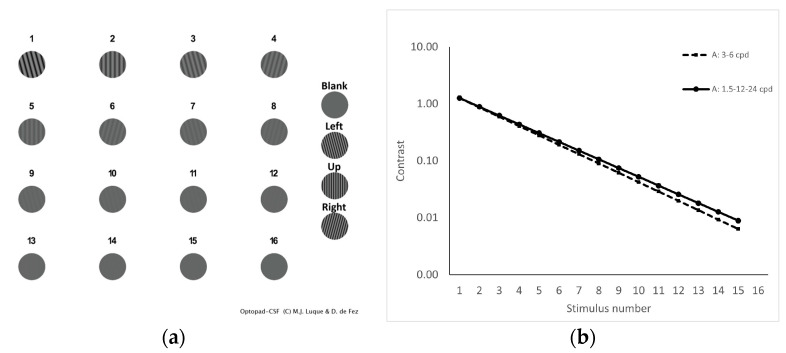
(**a**) Example of one of the five achromatic test plates of Optopad-CSF. Contrast decreases from left to right and from top to bottom, from the maximum generable stimulus (1) to zero contrast (16). Stimuli on the right column are references for the observer. (**b**) Cone contrast values for the fifteen stimuli in each plate of Optopad-CSF test, for the achromatic (A) cardinal directions. Note that step size depends on spatial frequency.

Achromatic CSF is measured by means of five plates, one for each spatial frequency evaluated (1.5, 3, 6, 12 and 24 cpd). Each plate contains a decreasing contrast series of sinusoidal gratings in 2-degree circular windows, arranged in a 4 × 4 grid against an achromatic background with the device’s maximum generable luminance (Figure 2a). Contrast values were chosen in logarithmic steps in cone contrast space, taking into account the frequency-dependent sensitivity of the visual system (Figure 2b). Contrasts for each color direction ranged from the maximum generable value to half the maximum non-detectable generable contrast, determined from preliminary trials with a set of six experienced observers in the 45–55 age range. The last stimulus of the series (plate’s stimulus 16) had zero amplitude. Grating orientation was randomly chosen among 3 possibilities (−15°, 0° and 15°).

The slides are presented to the patient one at a time, in a sense of increasing frequency. In each slide, the patient’s task was to indicate the orientation of the gratings of each of the 16 stimuli. The descending limit psychophysics method has been used, so thresholds are determined as the average contrast of the last hit and the first miss for each frequency. 

### 2.5. Statistical Analysis

The statistical analysis was performed using the SPSS statistical software version 28.0.0 for Windows (IBM SPSS Inc., Chicago, IL, USA). The normality of data distributions was confirmed by means of the Kolmogorov–Smirnov test. Differences between the measurements obtained in the right and left eyes were assessed by using either the paired Student’s *t* test or the Wilcoxon test, depending on whether the samples were normally distributed or not. For the comparison between the subgroups of healthy subjects aged 40 years or older and those below 40 years, the unpaired Student’s *t* test was used for analyzing the differences in normally distributed variables, whereas the Mann–Whitney test was used if the condition of normality did not hold. Either Pearson’s or Spearman’s correlation coefficient was calculated to investigate the relationship between different variables, depending on whether the variables correlated were normally distributed or not. A *p*-value < 0.05 was considered the criterion of statistical significance for all these tests.

As previously mentioned, the comparison between the results of the two devices used to measure the CSF must be approached with care due to their different designs and the measurement characteristics of each one. In the current study, this problem was addressed by two methods, based on a study by Koefoed [12]. The first method was calculating the area under the curve (AUC), which depends to a certain extent on the frequency range of each device as well as the maximum sensitivity value achieved in each one. The second method was calculating the value of the Index of Contrast Sensitivity (ICS), which allows comparison between different devices and measurement conditions, since it normalizes the results. The normalized ICS value is obtained by calculating the residuals with respect to the median of the normal population for each frequency and only in one eye (right eye). Differences were weighted according to the presumed clinical importance of each frequency. Thus, 6 cpd was given the highest weight (factor 3), whereas frequencies 3 and 12 cpd received factor 2, and the remaining test frequencies were not weighted. Bland and Altman analysis was used to analyze the level of interchangeability between CSF measurement methods in terms of ICS and AUC. For this purpose, the main differences between measurement methods and the limits of agreement (LoA) (±1.96 times the standard deviation of differences between methods) were calculated and plotted.

Finally, a range of normality was defined for the values obtained with the Optopad-CSF test, and it was calculated as the range contained between percentiles 5% and 95%.

## 3. Results

A total of 200 eyes of 100 healthy subjects with a mean age of 34.9 years (SD: 14.0, median: 31.5, range: 17 to 63 years) were evaluated. The sample consisted of 80 males (40.0%) and 120 females (60.0%). Table 1 summarizes the visual, refractive, and anatomical data of the sample evaluated, including the results of UDVA and CDVA, the refractive error of the eyes evaluated (sphere, cylinder, axis, and the vectorial notation of refraction), the measurement of the near visual function (uncorrected and distance-corrected near visual acuities), information about the eye (axial length and anterior chamber depth), corneal shape (keratometry, asphericity, horizontal corneal diameter), and pupil size. As shown, the only statistically significant differences between the right and left eyes were found in axial length (*p* = 0.023), K2 (*p* < 0.001), central corneal thickness (CCT) (*p* = 0.002), anterior chamber depth (ACD) (*p* = 0.042), white-to-white (WTW) corneal diameter (*p* = 0.011), and scotopic pupil size (*p* < 0.001). Table 2 summarizes the ocular aberrometric data of the sample evaluated, including all Zernike aberrometric terms defining the wavefront aberration error of the eye for two different pupil sizes, 3 and 5 mm. This analysis includes the measurement of high-order aberrations, such as primary coma or spherical aberrations. Statistically significant differences between fellow eyes were found in astigmatism (Z_2_^±2^) (*p* = 0.010) and secondary astigmatism (Z_4_^±2^) (*p* = 0.022) for a 3 mm pupil as well as in defocus (Z_2_^0^) (*p* = 0.003) for a 5 mm pupil.

### 3.1. Distance Contrast Sensitivity Data

**Figure 3:**
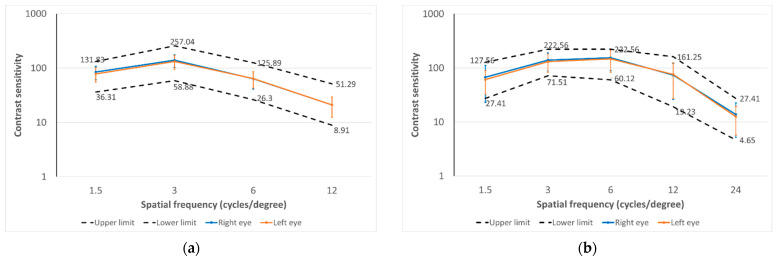
(**a**) Distance contrast sensitivity function outcomes measured with the CSV-1000E test in right and left eyes in the sample evaluated. The upper and lower limits of the range of normality provided by the manufacturer of this test is also displayed as dotted lines. (**b**) Near contrast sensitivity function outcomes measured with the Optopad-CSF test in the right and left eyes in the sample of healthy subjects evaluated. The upper and lower limits of the range of normality are also displayed as dotted lines. This range was calculated as the range contained between percentiles 5% and 95%.

Figure 3a shows the distance contrast sensitivity function outcomes measured with the CSV-1000E test in right and left eyes in the sample evaluated. The CSF represents, as previously mentioned, how the contrast sensitivity changes according to the spatial frequency evaluated, with the edges of the response within the normality for the healthy eye being represented by dotted lines. Significant differences were found in the distance CSF outcomes measured with the CSV-1000E test for the spatial frequencies of 3 (*p* = 0.008) and 6 cpd (*p* = 0.039). The mean absolute differences between fellow eyes in distance CSF were 13.69 (SD: 15.36; median: 0.00; range: 0.00 to 59.97), 21.76 (SD: 27.84; median: 0.00; range: 0.00 to 125.80), 12.79 (SD: 14.71; median: 11.93; range: 0.00 to 48.75), and 4.27 (SD: 5.16; median: 1.33; range: 0.00 to 17.70) for 3, 6, 12 and 18 cpd, respectively.

In the sample of right eyes, the distance CSF for the spatial frequency of 18 cpd was found to be significantly correlated with age (r = −0.376, *p* < 0.001), CDVA (r = −0.392, *p* < 0.001), DCNVA (r = −0.293, *p* = 0.003), HOA RMS (r = −0.230, *p* = 0.021), and trefoil (r = −0.288, *p* = 0.004) for the 3 mm pupil. Similarly, in the left eyes, the distance CSF for 18 cycles/degree was found to be correlated with age (r = −0.286, *p* = 0.004), CDVA (r = −0416, *p* < 0.001), DCNVA (r = −0.316, *p* = 0.001), HOA RMS (r = −0.244, *p* = 0.014), and trefoil (r = −0.221, *p* = 0.027) for the 3 mm pupil.

### 3.2. Near Contrast Sensitivity Outcomes

Figure 3b shows the near-contrast sensitivity function outcomes measured with the Optopad-CSF test in the right and left eyes in the sample evaluated. The upper and lower limits of the range of normality are displayed as dotted lines and were calculated as the percentiles 5% and 95%. Significant differences between fellow eyes were found in near CSF outcomes for the spatial frequency of 3 cpd (*p* = 0.004). Mean absolute differences between fellow eyes in distance CSF were 18.36 (SD: 30.58; median: 11.66; range: 0.00 to 229.83), 34.90 (SD: 68.91; median: 0.00; range: 0.00 to 395.07), 46.96 (SD: 80.41; median: 0.00; range: 0.00 to 324.95), 21.20 (SD: 20.32; median: 21.76; range: 0.00 to 85.72), and 4.84 (SD: 5.72; median: 3.43; range: 0.00 to 36.45) for 1.5, 3, 6, 12, and 24 cpd, respectively.

Table 3 summarizes the main correlations found between near CSF and different clinical variables. Specifically, it shows if some of the parameters defined in Table 1 showed a significant correlation with the near CSF values measured with Optopad-CSF. As shown, near CSF for all the spatial frequencies in both eyes showed a negative significant correlation with age, CDVA, and DCNVA. Likewise, near CSF for 6 cpd correlated significantly in both eyes with HOA RMS for the 3 mm pupil.

Regarding gender, no significant differences were found between males and females in the contrast sensitivity values obtained for the different spatial frequencies evaluated: 1.5 (*p* = 0.486), 3 (*p* = 0.160), 6 (*p* = 0.175), 12 (*p* = 0.159), and 24 cpd (*p* = 0.971).

### 3.3. Validation of Near Contrast Sensitivity Measures

The descriptive statistics showed that the mean AUC of the Optopad-CSF test (5.84) was twice that of the CSV-1000E test (2.76), as was also the case with the standard deviations. The Wilcoxon signed ranks test corroborates that this relationship of areas occurs in 96% of the cases.

The mean ICS analysis of Optopad-CSF (−0.019) and CSV-1000E (−0.075) showed similar values close to 0, although the standard deviation was higher in the case of Optopad-CSF since the range was approximately double. The mean difference obtained was much lower in our comparison than the results obtained by other authors [12,15], while the LoAs were similar or much lower in our case. The Wilcoxon signed rank test did not state that the measurements were statistically different (*p* = 0.3). However, the calculated residuals were high and equally distributed between positive and negative values.

**Figure 4:**
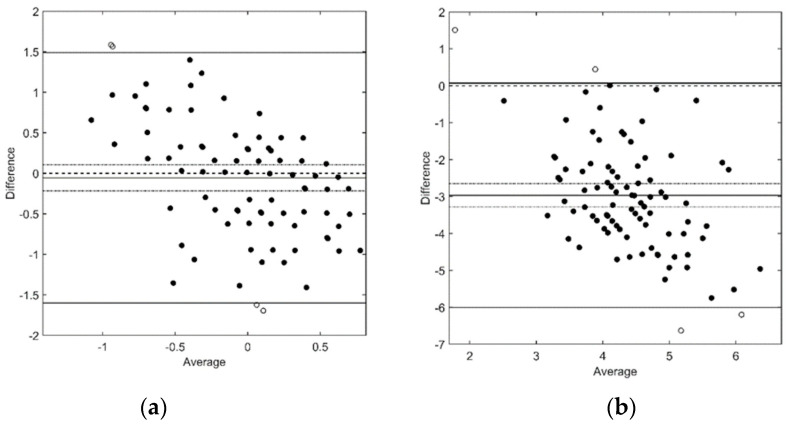
Bland–Altman diagram for (**a**) ICS and (**b**) AUC. Difference: CS-1000E—Optopad-CSF. Black circles are within mean difference ± 1.96 standard deviation of differences, whereas white circles were out of this range.

There was a weak but significant correlation between the Optopad-CSF and CSV-1000E ICS (r = 0.246, *p* < 0.02), but the correlation between the AUC in both devices was not significant (r = 0.001, *p* = 0.99). Given that differences in ICS and AUC between both CSF measuring systems followed a normal distribution, a Bland–Altman analysis was performed. The results of this analysis showed large LoAs for ICS (Figure 4a) and AUC (Figure 4b). The 95% confidence interval for the mean difference between measuring systems contained zero for ICS, and therefore the two devices may be considered to agree. However, the data showed a downwards slope, indicating that Optopad-CSF values were larger than CSV1000 values for the most sensitive patients, while the opposite occurred for the least sensitive patients. No agreement was found for AUC values. Although the LoA just enclosed zero, the 95% confidence interval for the median did not, indicating that larger AUC values were found with Optopad.

**Figure 5:**
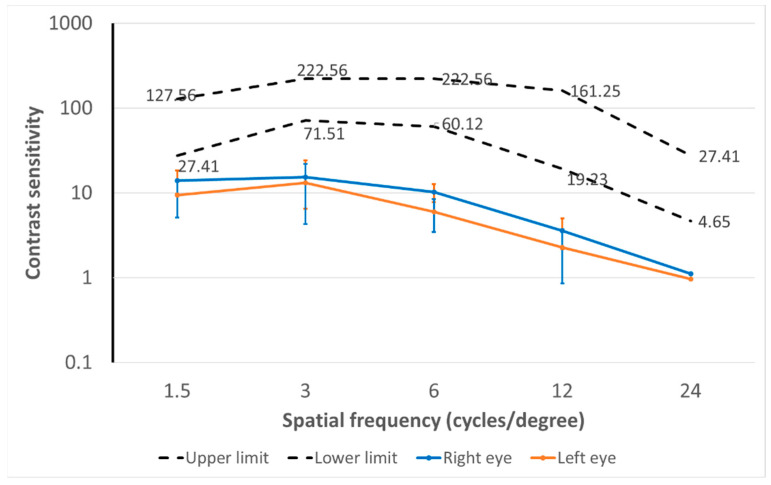
Near contrast sensitivity function outcomes measured with the Optopad-CSF test in the right and left eyes in the sample of subjects with bilateral cataracts evaluated. The upper and lower limits of the range of normality for reference are displayed as dotted lines.

A range of normality for the values obtained with the Optopad-CSF test was calculated as the range between percentiles 5% and 95%. For this purpose, data from right and left eyes were combined to obtain a single range valid for any eye. In Figure 5, the upper and lower limits of this range of normality were represented with dotted lines. Likewise, a range of normality using the same criterion was defined for the interocular difference in near CSF for each of the spatial frequencies evaluated: 1.5 (0.00–80.66), 3 (0.00 to 118.15), 6 (0.00 to 324.95), 12 (0.00 to 56.98), and 24 cpd (0.00 to 13.85).

These ranges were used to assess contrast sensitivity in a sample of 16 patients with bilateral cataracts with ages between 50 and 80 years, as displayed in Figure 5. The mean values were found to be out of the normal range for all the spatial frequencies evaluated. Furthermore, statistically significant differences in near CSF were found between healthy subjects and subjects with bilateral cataracts in right and left eye samples for all the spatial frequencies evaluated (all *p*-values < 0.001). When performing the analysis of these patients for distance CSF, the calculations revealed that for 18 cpd in both eyes, the mean values are within the normal range.

**Figure 6:**
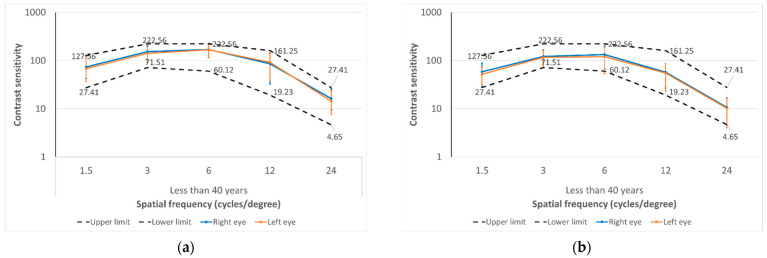
Near contrast sensitivity function outcomes measured with the Optopad-CSF test in the right and left eyes in two subgroups of eyes of the sample of healthy subjects evaluated defined according to age: (**a**) less than 40 years and (**b**) 40 years or more. The upper and lower limits of the range of normality for reference are displayed as dotted lines.

Finally, the near CSF measured with the Optopad-CSF test of healthy subjects aged 40 years or older and those below 40 years were compared. Significantly lower CS was found in the over-40-year-old group for all the spatial frequencies evaluated: 1.5 (right eye *p* = 0.008, left eye *p* = 0.002), 3 (right eye *p* = 0.001, left eye *p* = 0.003), 6 (right eye *p* = 0.011, left eye *p* < 0.001), 12 (right eye *p* = 0.001, left eye *p* < 0.001), and 18 cpd (both eyes *p* < 0.001) (Figure 6).

## 4. Discussion

Different tests for the measurement of the contrast sensitivity function can be used in clinical practice. Richman [11] compared numerous tests for contrast sensitivity measurement, using both letters and gratings, highlighting their advantages and disadvantages. The simplest and cheapest were letter tests, which did not give separate information for different frequencies but had high repeatability. Grating tests had limited repeatability, but they provided more information about the visual system by assessing different channels of spatial information. For this reason, these types of tests are the most commonly used, including the two tests for measuring the CSF compared in the current study. A healthy population was used in the first part of the validation of the Optopad-CSF test and its comparison with the CSV-1000E test. The condition of healthy eyes was confirmed after a complete visual, anatomical, and optical characterization of the sample, which showed a distribution of all these data within previously reported normal ranges [24,25,26].

Comparisons between different CSF measurement systems require considering the differences between viewing conditions and how the design of the stimuli used was conducted. The present study was based on contrast sensitivity function measurements using two tests based on sinusoidal stimuli. Measurements were taken at different distances (near: 40 cm and distance: 2.5 m), under different illumination conditions (Lmax: 460 cd/m^2^ for Optopad-CSF and 85 cd/m^2^ for CSV-1000E), for different spatial frequency values (Optopad-CSF: 1.5, 3, 6, 12, and 24 cpd; CSV-1000E: 3, 6, 12, and 18 cpd), and different contrast value sets for each frequency. In this sense, the maximum and minimum contrasts represented were chosen according to the responses of a normal population, and therefore, both tests did not contain too many stimuli. The difference in the contrast step size and the number of steps were also variables that differentiated the two devices. According to this, some differences could be expected, but both should be able to detect differences in the CSF with age and the very significant reduction of this function in cases of cataracts.

Other authors have performed comparisons between devices or measurement conditions using different methodologies for measuring the CSF [9,10,12,13,14,15,16,24]. Hitchcock [9] performed a study comparing a hand-held chart (FACT) with a vision tester (Optec 1000) analyzing the same spatial frequencies and found statistically significant differences in the values measured for both low and high frequencies. Franco [10] compared sensitivity values between the CSV-1000E and VCTS 6500, resulting in significant differences between the two devices. In this article, the measurements were taken under different observation conditions. The study by Dorr [27] was based on custom-designed CRT- and tablet-based tests, demonstrating that contrast sensitivity function (CSF) assessment on a mobile device was indistinguishable from that obtained with specialized laboratory equipment. In this case, the authors designed comparable stimuli for both tests, indirectly confirming the dependence of test parameters and observation conditions obtained in other comparisons.

In many cases, authors compared tests designed with different parameters by calculating the areas under the curve or by using the method proposed by Wachler and Krueger [28], which was based on normalizing the sensitivity results. For example, Gil [16] measured the CSF with the CSV-1000 E and VCTS 6000 systems; Altinbay [15] compared the CSV-1000E and Mon Pack 3 from Metrovision; Haughom [14] evaluated the Optec 6500⁄FACT system under different illumination conditions; and Koefoed [12] compared the CSV-1000E and FACT. In our study, the methods proposed by these authors were used, with the same criteria defined by Koefoed to normalize the contrast sensitivity values, and, as an alternative method, the area under the curve was also compared. The AUC analysis did not show interchangeability between CSV-1000E and Optopad-CSF, while the ICS analysis indicated that the results of both tests were correlated. The differences found between the ICS of both tests should be assessed based on clinical significance, a question that is still open [12,14,15].

Regarding the ceiling effect, which is common in contrast sensitivity tests and in particular with the CSV-1000E test [12], our results corroborated this phenomenon. Approximately 24% of patients evaluated with the CSV-1000E test selected the stimulus with the lowest contrast at two or more frequencies. However, for the Optopad-CSF test, the selection of the stimulus with the lowest contrast at two or more frequencies only occurred in one case. Specifically, this type of selection occurred more frequently for the spatial frequency of 6 cpd, being present in 3% of the patients. This is, from our point of view, a great advantage of the Optopad test.

As provided for the CSV-1000E test, a range of normality was defined for the Optopad-CSF test for the five spatial frequencies evaluated. This range is especially useful in clinical practice for defining which cases are suspected of being anomalous. Future studies including larger sample sizes should be performed to refine this range of normality and define even more specific ranges by age subgroups. It should be considered that, as happened with many other contrast sensitivity tests, a correlation has been found between contrast sensitivity values and age.

Contrast sensitivity analysis with the Optopad-CSF test has shown in our sample promising results. Specifically, significant differences were found with the test between healthy subjects under and over 40 years of age, corroborating that sensitivity declined with age. This phenomenon was detected and characterized in detail by Sia et al. [24] using the CSV-1000E test. Therefore, the Optopad-CSF test also identified these age-related changes in contrast sensitivity for all the spatial frequencies evaluated. Besides this, the Optopad-CSF test could detect the reduction in contrast sensitivity in those eyes with bilateral cataracts, as could be expected according to the negative impact of loss of crystalline lens transparency on visual function [24]. The mean results of the CSV-1000E test in the group of patients with bilateral cataracts in our study only showed abnormal behavior for medium and low spatial frequencies, suggesting some potential level of limitation of the diagnostic ability of this test for high spatial frequencies. When evaluating the ICS in this pathological population, an average of −5.81 with a SD of 1.09 was obtained, outside the range of normal values obtained in our study. Future research shall include functional losses related to damage in the visual pathway, which may result in out-of-normal-bound values in single spatial frequencies but also in changes in the shape of the CSF [29] or in the inter-ocular CSF difference if the pathology does not affect equally both eyes. Different computation techniques may be used to detect these changes, including machine learning.

Although differences between the CSV1000 and Optopad could be explained by the physical differences between stimuli, including mean luminance and contrast step size, the possible influence of the characteristics of the light source (uniformity and polarization) must be further studied. Alternative reasons for the different behavior observed for low- and high-sensitivity subjects in the Bland-Altman plots must also be explored, including the possible influence of retinal illumination and scatter linked with iris color [30], pupil size, and the density of macular pigment.

## 5. Conclusions

The comparison of contrast sensitivity results between one of the most widely used tests, the CSV-1000E test, and the Optopad-CSF test shows that contrast sensitivity measures are not interchangeable. This result was as expected due to the differences between the two tests: distance, spatial frequencies, illumination, stimulus size, contrast steps, and psychophysical method. Normalizing the sensitivity values and calculating the ICS parameter, a correlation was found between the results of both tests. Considering additionally that contrast sensitivity measurements obtained with the Optopad-CSF test correlate with age and that the test detects without any difficulty those cases with a sensitivity reduction due to the presence of cataracts, it can be stated that the Optopad-CSF test provides valid measures for its clinical use.

Both tests are fast and do not require external illumination, but Optopad-CSF has the advantage of being portable. In addition, it has been designed using five spatial frequencies (one more than the CSV-1000E) and with sixteen contrast steps (compared to eight in the other test). The evaluation of contrast sensitivity is much more precise and would increase the diagnostic accuracy, especially for high spatial frequencies. In addition, it does not have the ceiling effect that is clearly present in the CSV-1000E test. Another great advantage is that, as it is easily programmable, the intensity values of the adaptation, spatial frequencies, contrasts, and the type of stimulus can be changed. Our test is prepared to be modified according to the need of the study.

On the other hand, as we have indicated in the Methodology section, the test has not only been designed in the achromatic version, but also red–green and blue–yellow chromatic tests have also been implemented, which are currently in the study phase.

The results of this contrast sensitivity study, together with those previously obtained in chromatic discrimination, support, from our point of view, the great potential of the Optopad Project.

## Figures and Tables

**Table 1:** Summary of the visual, refractive and anatomical data of the sample evaluated.

Mean (SD) Median (Range)	Right Eye	Left Eye	*p*-Value
LogMAR UDVA	0.43 (0.71) 0.15 (−0.18 to 2.00)	0.42 (0.69) 0.15 (−0.18 to 2.00)	0.780
LogMAR UNVA	0.32 (0.41) 0.20 (−0.10 to 2.00)	0.30 (0.39) 0.10 (−0.10 to 2.00)	0.055
Sphere (D)	−0.85 (2.56) −0.25 (−9.25 to 5.25)	−0.70 (2.59) 0.00 (−10.00 to 5.25)	0.074
Cylinder (D)	−0.53 (0.76) −0.25 (−4.25 to 0.00)	−0.55 (0.71) −0.25 (−3.00 to 0.00)	0.263
SE (D)	−1.11 (2.62) −0.38 (−10.37 to 5.25)	−0.97 (2.65) −0.25 (−11.12 to 5.25)	0.147
J_0_ (D)	0.06 (0.40) 0.00 (−1.06 to 1.63)	0.08 (0.39) 0.00 (−0.86 to 1.49)	0.204
J_45_ (D)	0.00 (0.23) 0.00 (−0.74 to 1.37)	−0.02 (0.21) 0.00 (−0.72 to 0.72)	0.691
B (D)	1.91 (2.15) 1.03 (0.00 to 10.44)	1.91 (2.12) 1.03 (0.00 to 11.18)	0.970
LogMAR CDVA	−0.12 (0.05) −0.10 (−0.18 to 0.10)	−0.12 (0.06) −0.10 (−0.18 to 0.05)	0.159
LogMAR DCNVA	0.13 (0.22) 0.00 (−0.10 to 0.92)	0.13 (0.21) 0.00 (−0.10 to 0.80)	0.447
Near addition (D)	0.70 (0.92) 0.00 (0.00 to 3.00)	0.70 (0.92) 0.00 (0.00 to 3.00)	0.999
LogMAR CNVA	−0.01 (0.03) 0.00 (−0.10 to 0.00)	−0.01 (0.03) 0.00 (−0.10 to 0.10)	0.317
AXL (mm)	23.97 (1.21) 23.80 (21.63 to 27.66)	23.88 (1.23) 23.79 (21.50 to 27.77)	0.023
K1 (D)	7.95 (0.30) 7.94 (7.32 to 9.12)	7.92 (0.29) 7.92 (7.31 to 8.82)	0.649
K2 (D)	7.80 (0.32) 7.83 (7.19 to 9.03)	7.76 (0.28) 7.74 (7.18 to 8.49)	<0.001
Q	−0.19 (0.17) −0.19 (−0.73 to 0.71)	−0.23 (0.17) −0.19 (−0.69 to 0.18)	0.056
CCT (micrometers)	557.96 (35.79) 556.00 (471 to 638)	560.64 (36.03) 559.00 (468 to 648)	0.002
ACD (mm)	2.93 (0.40) 3.00 (1.56 to 3.73)	2.94 (0.42) 3.01 (1.64 to 3.74)	0.042
WTW (mm)	12.09 (0.53) 12.08 (10.79 to 13.19)	12.03 (0.55) 12.05 (10.42 to 13.33)	0.011
Scotopic pupil size (mm)	5.61 (0.97) 5.65 (2.90 to 7.90)	5.87 (1.13) 5.90 (3.50 to 8.50)	<0.001
Photopic pupil size (mm)	2.89 (0.39) 2.90 (1.90 to 3.90)	2.94 (0.52) 2.80 (1.70 to 5.40)	0.187

Abbreviations: SD, standard deviation; D, diopters; UDVA, uncorrected distance visual acuity; UNVA, uncorrected near visual acuity; SE, spherical equivalent; J_0_ and J_45_: power vector components of astigmatism; B, overall blur strength; CDVA, corrected distance visual acuity; DCNVA, distance-corrected near visual acuity; CNVA, corrected near visual acuity; AXL, axial length; K1, flattest keratometric reading; K2, steepest keratometric reading; Q, asphericity of the anterior corneal surface; CCT, central corneal thickness; ACD, anterior chamber depth; WTW, white-to-white corneal diameter.

**Table 2:** Summary of the ocular aberrometric data of the sample evaluated for 3 and 5-mm pupil aperture.

Mean (SD) Median (Range)	Right Eye	Left Eye	*p*-Value
Z_2_^0^ (µm) 3 mm	0.37 (0.89) 0.23 (−1.96 to 3.24)	0.32 (0.91) 0.16 (−1.83 to 3.54)	0.132
Z_2_^±2^ (µm) 3 mm	0.18 (0.16) 0.15 (0.01 to 1.06)	0.21 (0.17) 0.17 (0.00 to 0.85)	0.010
LOA RMS (µm) 3 mm	0.73 (0.67) 0.45 (0.06 to 3.25)	0.74 (0.67) 0.46 (0.04 to 3.57)	0.657
HOA RMS (µm) 3 mm	0.06 (0.03) 0.05 (0.01 to 0.19)	0.06 (0.04) 0.06 (0.02 to 0.39)	0.449
Z_3_^±1^ (µm) 3 mm	0.03 (0.02) 0.03 (0.00 to 0.10)	0.04 (0.03) 0.03 (0.00 to 0.26)	0.518
Z_3_^±3^ (µm) 3 mm	0.04 (0.03) 0.03 (0.00 to 0.17)	0.04 (0.03) 0.03 (0.00 to 0.22)	0.730
Z_4_^0^ (µm) 3 mm	0.00 (0.01) 0.00 (−0.04 to 0.04)	0.00 (0.02) 0.00 (−0.06 to 0.04)	0.658
Z_4_^±2^ (µm) 3 mm	0.01 (0.01) 0.01 (0.00 to 0.04)	0.01 (0.01) 0.01 (0.00 to 0.13)	0.022
Z_2_^0^ (µm) 5 mm	0.94 (1.94) 0.60 (−3.18 to 7.53)	0.76 (1.86) 0.35 (−4.42 to 6.38)	0.003
Z_2_^±2^ (µm) 5 mm	0.40 (0.37) 0.29 (0.01 to 2.07)	0.43 (0.39) 0.32 (0.02 to 2.43)	0.183
LOA RMS (µm) 5 mm	1.60 (1.55) 1.00 (0.11 to 7.58)	1.53 (1.42) 0.88 (0.07 to 6.42)	0.143
HOA RMS (µm) 5 mm	0.17 (0.08) 0.15 (0.04 to 0.48)	0.17 (0.09) 0.15 (0.04 to 0.52)	0.295
Z_3_^±1^ (µm) 5 mm	0.09 (0.07) 0.08 (0.00 to 0.35)	0.10 (0.07) 0.09 (0.00 to 0.44)	0.547
Z_3_^±3^ (µm) 5 mm	0.09 (0.06) 0.08 (0.00 to 0.35)	0.09 (0.07) 0.08 (0.00 to 0.30)	0.285
Z_4_^0^ (µm) 5 mm	0.03 (0.06) 0.02 (−0.18 to 0.18)	0.02 (0.06) 0.02 (−0.19 to 0.23)	0.638
Z_4_^±2^ (µm) 5 mm	0.03 (0.02) 0.03 (0.00 to 0.13)	0.03 (0.02) 0.03 (0.00 to 0.16)	0.484

Abbreviations: SD, standard deviation; D, diopters; LOA, low order aberrations; RMS, root mean square; HOA, high-order aberrations; Z_2_^0^, defocus; Z_2_^±2^, astigmatism; Z_3_^±1^, astigmatism, primary coma; Z_3_^±3^, trefoil; Z_4_^0^, primary spherical aberration; Z_4_^±2^, secondary astigmatism.

**Table 3:** Main correlations found between near contrast sensitivity function measured with the Optopad-CSF test and different clinical variables in both right and left eyes sample.

Coefficient of Correlation (*p*-Value)	Right Eye	Left Eye
Near CSF 1.5 cpd	Age r = −0.257, *p* = 0.010 CDVA r = −0.332, *p* < 0.001 DCNVA r = −0.298, *p* = 0.003 HOA RMS 3 mm r = −0.251, *p* = 0.012 Z_4_^±2^ 3 mm r = −0.229, *p* = 0.022 Z_3_^±3^ 3 mm r = −0.256, *p* = 0.010	Age r = −0.316, *p* = 0.001 CDVA r = −0.285, *p* = 0.004 DCNVA r = −0.332, *p* < 0.001
Near CSF 3 cpd	Age r = −0.315, *p* = 0.001 CDVA r = −0.296, *p* = 0.003 DCNVA r = −0.330, *p* < 0.001 Photopic pupil size r = 0.206, *p* = 0.040	Age r = −0.312, *p* = 0.002 CDVA r = −0.247, *p* = 0.013 DCNVA r = −0.368, *p* < 0.001 Distance CS 3 cycles/° r = 0.218, *p* = 0.029
Near CSF 6 cpd	Age r = −0.313, *p* = 0.001 CDVA r = −0.411, *p* < 0.001 DCNVA r = −0.302, *p* = 0.002 HOA RMS 3 mm r = −0.231, *p* = 0.021 Photopic pupil size r = 0.245, *p* = 0.014	Age r = −0.392, *p* < 0.001 CDVA r = −0.381, *p* < 0.001 DCNVA r = −0.400, *p* < 0.001 HOA RMS 3 mm r = −0.352, *p* < 0.001 Z_3_^±1^ 3 mm r = −0.267, *p* = 0.007 Z_3_^±1^ 5 mm r = −0.222, *p* = 0.026
Near CSF 12 cpd	Age r = −0.309, *p* = 0.002 CDVA r = −0.512, *p* < 0.001 DCNVA r = −0.363, *p* < 0.001 Photopic pupil size r = 0.287, *p* = 0.004 Distance CS 12 cycles/° r = 0.271, *p* = 0.006	Age r = −0.395, *p* < 0.001 CDVA r = −0.441, *p* < 0.001 DCNVA r = −0.432, *p* < 0.001 HOA RMS 3 mm r = −0.290, *p* = 0.003
Near CSF 24 cpd	Age r = −0.349, *p* < 0.001 CDVA r = −0.389, *p* < 0.001 DCNVA r = −0.320, *p* = 0.001 Photopic pupil size r = 0.208, *p* = 0.038	Age r = −0.258, *p* = 0.009 CDVA r = −0.348, *p* < 0.001 DCNVA r = −0.326, *p* < 0.001

Abbreviations: SD, standard deviation; D, diopters; CSF, contrast sensitivity function¸ HOA, high order aberrations; Z_4_^±2^, secondary astigmatism; Z_3_^±1^, primary coma; Z_3_^±3^, trefoil, CDVA, corrected distance visual acuity; DCNVA, distance-corrected near visual acuity.

## Data Availability

Data available on request from the authors.
